# Effects of the Combined Application of Trimethylated Chitosan and Carbodiimide on the Biostability and Antibacterial Activity of Dentin Collagen Matrix

**DOI:** 10.3390/polym14153166

**Published:** 2022-08-03

**Authors:** Xiangyao Wang, Qilin Li, Haibo Lu, Zhuo Liu, Yaxin Wu, Jing Mao, Shiqiang Gong

**Affiliations:** 1 Department of Stomatology, Tongji Hospital, Tongji Medical College, Huazhong University of Science and Technology, Wuhan 430030, China; m202076256@hust.edu.cn (X.W.); qilinli24@foxmail.com (Q.L.); luhaibo023@163.com (H.L.); d202081835@hust.edu.cn (Z.L.); yaxinwu@163.com (Y.W.); 2 School of Stomatology, Tongji Medical College, Huazhong University of Science and Technology, Wuhan 430030, China; 3 Hubei Province Key Laboratory of Oral and Maxillofacial Development and Regeneration, Wuhan 430022, China

**Keywords:** dentin collagen, TMC, EDC, cross-linking, antibacterial activity

## Abstract

The structural integrity of a dentin matrix that has been demineralized by the clinical use of etchants or calcium-depleting endodontic irrigants, such as endodontic ethylenediaminetetraacetic acid (EDTA), is often deteriorated due to the collagenolytic activities of reactivated endogenous enzymes as well as the infiltration of extrinsic bacteria. Therefore, the biomodification of dentin collagen with improved stability and antibacterial activity holds great promise in conservative dentistry. The purpose of this study was to evaluate the effects of the combined application of trimethylated chitosan (TMC) and 1-ethyl-3-[3-dimethylaminopropyl]carbodiimide hydrochloride (EDC) on the biostability and antibacterial activity of the demineralized dentin collagen matrix. The morphological changes in the collagen matrix were observed by scanning electron microscopy (SEM), the amount of TMC adsorbed on the collagen surface was detected by X-ray photoelectron spectroscopy, and the elastic modulus was measured by a three-point bending device. Dry weight loss and amino acid release were detected to evaluate its anti-collagenase degradation performance. The antibacterial performance was detected by confocal microscopy. The TMC-treated group had less collagen space and a more compact collagen arrangement, while the untreated group had a looser collagen arrangement. The combined application of TMC and EDC can increase the elastic modulus, reduce the loss of elastic modulus, and result in good antibacterial performance. The current study proved that a dentin collagen matrix biomodified by TMC and EDC showed improved biodegradation resistance and antibacterial activities.

## 1. Introduction

Despite substantial progress achieved in resin-based materials in restorative dentistry, the adhesive interface remains the Achille’s heel of the restoration. Clinically, dentin usually undergoes demineralization when it is conditioned by etchants (e.g., phosphoric acid) or calcium-chelating endodontic irrigants (e.g., EDTA) to create a resin-dentin bonding interface. The collagen matrix in the resin-dentin bonding interface is prone to destruction when dentin is depleted of minerals and leaves the collagen matrix denuded [1,2,3,4].

The micromechanical interlocking junctions between resin and collagen fibrils in the adhesive interface are the structural foundation (i.e., hybrid layer) of dentin bonding and are responsible for the long-term performance of restoration. However, the dentin demineralization process initiated by phosphoric acid or EDTA results in the loss of unbound and bound water of collagen, which may lead to the collapse of the collagen matrix [5]. This ultimately impairs the infiltration of resin monomers into the collagen fibrils. The denuded collagen in the hybrid layer that has not been embedded in the resin is vulnerable to biodegradation over time [6,7,8]. The main cause of collagen degradation is endogenous proteases. These enzymes include matrix metalloproteinases (MMPs) and cysteine cathepsins. They are calcium- and zinc-dependent proteases that are present in the inactive state of dentin collagen. When dentin is demineralized by acid etching, the acidic environment activates endogenous enzymes, converting them to the active state [9,10], which starts to degrade collagen. Another threat to the longevity of the adhesive interface is the ingress of bacteria and its by-product through the interfacial gaps. The bacterial cells are usually left on the remaining hard tissues during cavity preparation under the guidelines of so-called minimally invasive dentistry or root canal treatment, which was shown to be unable to eradicate all the bacteria in the canal system. Conferring dentin collagen with concomitant biodegradation resistance and antibacterial activity through dentin biomodification would be attractive [11,12]

**Figure 1:**
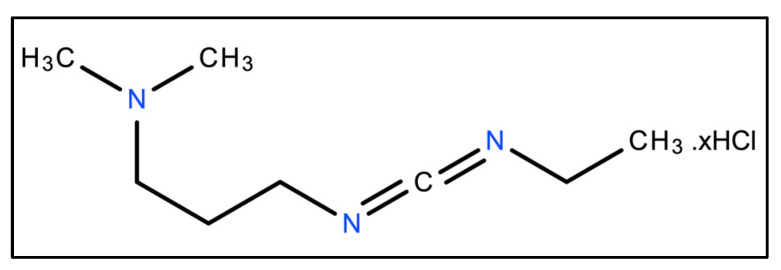
Chemical structure of 1-ethyl-3-[3-dimethylaminopro-pyl]carbodiimide hydrochloride (EDC).

The cross-linking method generally incorporates an intermediate molecule as a part of the cross-link component. The covalent cross-links could be formed by exogenous cross-links (e.g., riboflavin [13] and glutaraldehyde [14,15]), which inhibit MMP active sites from maintaining the stability and longevity of resin-dentin bonds [16,17]. However, these exogenous compounds have disadvantages, such as the toxicity of glutaraldehyde to mammalian cells [18] and the uncertainty of riboflavin’s long-term effect [19], which limits their use in the clinic. In addition, zero-length linkers are used as cross-linker agents [20], and 1-ethyl-3-[3-dimethylaminopropyl]carbodiimide hydrochloride (EDC) is one of the most studied collagen cross-linking agents and is nontoxic [14,21]. It enhances cross-linking by activating the carboxyl groups of glutamic and aspartic acids in proteins, which then react with the *ε*-amino groups present in the protein molecules, leading to the production of covalent cross-links [22]. It is also called a zero-length cross-linker because it directly promotes the cross-linking of adjacent protein chains, with the only product being urea [23,24]. Additionally, EDC (Figure 1) can also bind to glutamate and aspartate residues of endogenous proteases and thus inhibit their activities [25]. Thus, the performance of EDC has been shown to be superior in enhancing collagen strength and inhibiting the activities of proteases.

Chitosan is a polysaccharide obtained by the deacetylation of chitin, which is widely found in nature. Chitosan has a large number of free carboxyl and amino groups, which can produce microfibrillar arrangements in the protein matrix to form ionic complexes [26] and thus can be applied to cross-link collagen to improve its biological and mechanical properties, which provides advantages in tissue engineering applications [27]. Additionally, chitosan possesses resistance to bacterial collagenase degradation, inhibits bacterial adhesion to dentin [28], and has excellent biocompatibility [29]. However, the drawback that limits its application is that chitosan has poor aqueous solubility and its antibacterial effect is pH-dependent. A chitosan derivative obtained from the quaternization of chitosan’s primary amine groups has been shown to be soluble under physiological conditions [30]. Moreover, *N*,*N*,*N*-Trimethyl chitosan (TMC) bearing permanent quaternary moieties (i.e., quaternary ammonium) has shown improved antibacterial activities [31]. This makes TMC an excellent candidate in the scenarios that use quaternary ammonium compounds as antimicrobial agents in restorative dentistry [32,33,34]

In this work, we aimed to obtain a biomodified dentin collagen matrix with concomitant biodegradation resistance and antibacterial activity through the combined application of TMC and EDC.

## 2. Materials and Methods

### 2.1. Tooth Collection

With the consent of the Ethics Committee of Tongji Hospital, Tongji Medical College, Huazhong University of Science and Technology, 163 healthy molar teeth were extracted from patients attending Tongji Hospital and stored at 4 °C.

### 2.2. Dentin Sample Preparation

The dentin of 103 extracted healthy molar crowns was cut into 4 × 1 mm slices using a hard tissue slicer (Shenyang Kejing Automation Equipment Co., Ltd., SYJ-150, Shenyang, China) to observe the morphological structure, TMC adsorption content, and antibacterial assay; 60 extracted healthy teeth were cut into 6 × 1.5 × 1 mm strips for collagen degradation and elastic modulus assays.

First, we synthesized *N*,*N*,*N*-trimethyl chitosan (TMC), which was synthesized by a two-way pathway of trimethylated *N*,*N*-trimethyl chitosan (DMC) under relatively mild reaction conditions to avoid *O*-methylation, according to a previous report [31]. Briefly, 10 g of chitosan (MW = 100 kDa, degree of deacetylation ≥95%, Shanghai Aladdin Biochemical Technology Co., Ltd., Shanghai, China) was transferred into a 500 mL flask before adding 30 mL of formic acid, 40 mL of formaldehyde (37% stabilized with methanol), and 180 mL of distilled water and then reacted at 70 °C for 118 h under magnetic stirring. Then, the viscous solution was evaporated under reduced pressure, and 1 M NaOH aqueous solution was used to increase the pH to 12 to initiate gelation. The aforementioned gel was washed with deionized water to remove impurities, dissolved in HCl aqueous solution (pH 4.0), dialyzed against deionized water for 3 days, and lyophilized. Thus, *N*,*N*-dimethyl chitosan (DMC) was obtained. A total of 250 mg of DMC was dissolved in 40 mL of deionized water, and the pH was adjusted by NaOH to 11, at which gelation occurred. Then, the gel was rinsed thoroughly with water and acetone. Afterward, DMC was suspended in 50 mL NMP and 2 mL methyl iodide. The dispersion was stirred at 70 °C for the desired time and subsequently dropped into a 1:1 (*v*/*v*) ethanol/diethyl ether mixture to precipitate the product. To perform ion exchange, the precipitate was dialyzed against 1% NaCl aqueous solution for 3 days by changing the buffer twice a day and deionized water for another 3 days, and then lyophilized to obtain TMC. The chemicals used in this section were purchased from Sigma-Aldrich unless otherwise stated.

The cut samples were placed in 20 mL of 10% phosphoric acid solution (Sinopharm Group Chemical Reagent Co., Ltd., Shanghai, China) and demineralized by acid etching at 200 rpm for 48 h on a constant-temperature shaker and replaced with fresh phosphoric acid solution after 24 h for complete demineralization. The demineralized dentin pieces were rinsed with plenty of deionized water to remove the phosphoric acid from the surface, dried, and randomly divided into 5 groups (one extra dentin piece was treated with deionized water for aseptic testing of the antimicrobial assay):

Group A: deionized water
Group B: 0.5 M EDC (Wuhan Corey Biotechnology Co., Ltd., Wuhan, China) solution (pH 4–6)
Group C: 1 mg/mL TMC + 0.5 M EDC
Group D: 5 mg/mL TMC + 0.5 M EDC
Group E: 10 mg/mL TMC + 0.5 M EDC

For each of the reagents, the reaction time was 15 min. The unreacted reagents were then washed away by rinsing with deionized water before applying the next one.

### 2.3. Morphological and Structural Changes after Dentin Collagen Cros-Slinking

A total of 20 dentin slices were randomly selected from each group (*n* = 4), were dehydrated by an ethanol (Tianjin Hengxing Chemical Reagent Manufacturing Co., Ltd., Tianjin, China) gradient, and then fixed by drying with hexamethyldisilylamine (Shanghai Maclean Biochemical Technology Co., Ltd., Shanghai, China) for 15 min and then dried for 30 min by a vacuum dryer (Shanghai Junyi Instruments & Equipment Co., Ltd., Shanghai, China). The slices were then coated with gold using a gold sputter coater (JFC-1300, Jeol, Tokyo, Japan) for 40 s and observed by SEM (SU8010, Hitachi, Japan).

### 2.4. TMC Surface Content Test

A total of 8 prepared dentin slices from each group (except the EDC group), 32 slices in total, were randomly selected and dried in a vacuum dryer for 6 h. The change in elemental content of the collagen surface was then measured using a Thermo Fisher ESCALAB 250Xi photoelectron spectrometer with a monochromated Al Ka excitation source to determine the amount of TMC adsorbed on the dentin surface.

### 2.5. Dentin Collagen Biodegradation Test

In each group, 4 dentin strips were randomly selected, 20 strips in total, and placed in a vacuum desiccator for drying. The samples were dried for 6 h, and then the weight of each sample was measured. After that, the samples were transferred into EP tubes containing 320 µL of type I collagenase (Wuhan Corey Biotechnology Co., Ltd.) and degraded at 37 °C for 48 h. The samples were removed, rinsed with deionized water, and dried under vacuum for 6 h, and their weight was measured again to calculate the percentage of dry weight loss. The enzymatic solution was collected, 1 mL of hydrochloric acid solution (Xinyang City Chemical Reagent Factory, Xinyang, China) was added to each tube, and the hydroxyproline (HYP) was completely cleaved in a 95 °C water bath for 5 h. The pH was adjusted to 6.0–6.8, and the volume was fixed to 10 mL with deionized water. One milliliter of the solution was taken according to the instructions of the hydroxyproline kit (Nanjing Jiancheng Institute of Biological Engineering, Nanjing, China), and the absorbance of the solution was measured at 550 nm under an enzymatic labeler (Megagu Molecular Instruments Co., Ltd. SpectraMax i3, Shanghai, China) to calculate the amount of hydroxyproline released; thus, the degradation of dentin collagen was obtained.

### 2.6. Mechanical Properties

A total of 8 randomly selected prepared dentin strips from each group, 40 in total, were used to measure the elastic modulus of the reacted samples using a three-point bending device. We termed it the three-point bending method since the base platform included two metal sheets parallel to one another, 5 mm apart, on which dentin strips could be placed. A probe was also placed above the base platform to measure the pressure. The dentin strips were fully submerged in deionized water, with continuous application of pressure, with each displacement corresponding to the applied pressure. Each sample yields a linear stress-strain curve that, after cross-linking, provides the instantaneous modulus of elasticity. To prevent damaging the collagen and overstretching the dentin, this should be performed at 10% of its strain range. The samples were then put into EP tubes with 320 L of collagenase type I, where they were subsequently broken down for 48 h at 37 °C. The modulus of elasticity was then measured again after degradation.

### 2.7. Antimicrobial Activities

The −80° frozen Enterococcus faecalis (ATCC-29212) was placed in a 37 °C water bath for 1 min to allow the bacteria to fully recover. The filter paper containing the bacteria was coated on a brain heart agar plate (Biddy Medical Devices Shanghai Co., Ltd., Shanghai, China) and placed in a 37 °C thermostat (Ningbo Jiuxing Medical Equipment Co., Ltd., Ningbo, China) for 24 h. A colony of the bacteria was selected and fully dissolved in 20 mL of brain heart infusion (BHI) (Biddy Medical Devices Shanghai Co., Ltd.) and placed in a 37 °C shaker at 200 rpm for 12 h to amplify the bacteria, and the McFarland concentration was adjusted to 1 using a turbidimeter (WGZ-2XJ Shanghai Xinrui Instruments Co., Ltd., Shanghai, China). The final concentration was determined to be 1 × 108 CFU/mL.

Fifty well-prepared samples were placed in 96-well plates, with ten samples per group. The samples were sterilized by ultraviolet (UV) light for 30 min. Then, 2 µL of bacterial suspension was added to the surface of each sample and left for 10 min to completely penetrate the dentin. Then, 198 µL of brain heart infusion medium was added separately, in which the DW group added another sample for aseptic testing without bacteria, and only 200 µL of BHI was added to determine the aseptic effect and incubated together in a 37 °C thermostat for 48 h. Afterward, the samples were rinsed with plenty of deionized water to remove the unattached bacteria.

Afterward, 5 samples from each group were randomly selected and transferred into 1.5 mL EP tubes, and 200 µL of brain heart infusion medium was added and shaken for 1 min with an ultrasonic shaker to detach the bacteria from the collagen matrix. The dentin slices were removed from the EP tube, and the remaining *E. faecalis* solution was mixed and diluted in 5 concentration gradients with 0.01 M PBS. For each sample, 20 µL of each of the 6 concentration groups was aspirated and applied to a brain heart infusion agar plate and incubated at 37 °C for 48 h. Afterward, the agar plate was removed, and the number of colonies on the plate was counted using an automatic colony counter (Iount 10 Hangzhou Xuncui Technology Co., Ltd., Hangzhou, China), and the concentration of the bacterial suspension was calculated by including the plates in the range 30–300 colonies.

The remaining 5 samples from each group were stained with LIVE/DEAD Baclight (L7012 Dye Thermo Fisher Scientific, Waltham, MA, USA) dye for 30 min, rinsed with 100 mM PBS, placed under a confocal laser scanning microscope, and observed with red and green fluorescence, with red representing dead bacteria and green representing surviving bacteria. The ratio of the two was observed to assess the estimated antimicrobial performance of collagen after treatment with different reagents.

### 2.8. Statistical Analysis

The data of percent dry weight loss, elastic modulus, and degradation percentage of elastic modulus and hydroxyproline content were evaluated with Shapiro–Wilk and Kolmogorov–Smirnov tests (SPSS Statistics 26.0, IBM Corporation, Armonk, NY, USA) to assess whether the data followed a normal distribution. Levene’s test was performed to determine the homogeneity of variance. Statistical significance was determined by a one-way analysis of variance, followed by Turkey’s multiple comparison (Graph Pad Prism, Version 9, Graph Pad Software Inc., La Jolla, San Diego, CA, USA). The data of the release of HYP and colony-forming units of bacteria were analyzed using non-parametric tests (Kruskal–Wallis test). In all cases, the global significance level was 5%. Power analysis was conducted to determine the power of each experiment and to project the effect size as 0.4 and the significance level as 0.05.

## 3. Results

### 3.1. Morphological and Structural Changes

**Figure 2:**
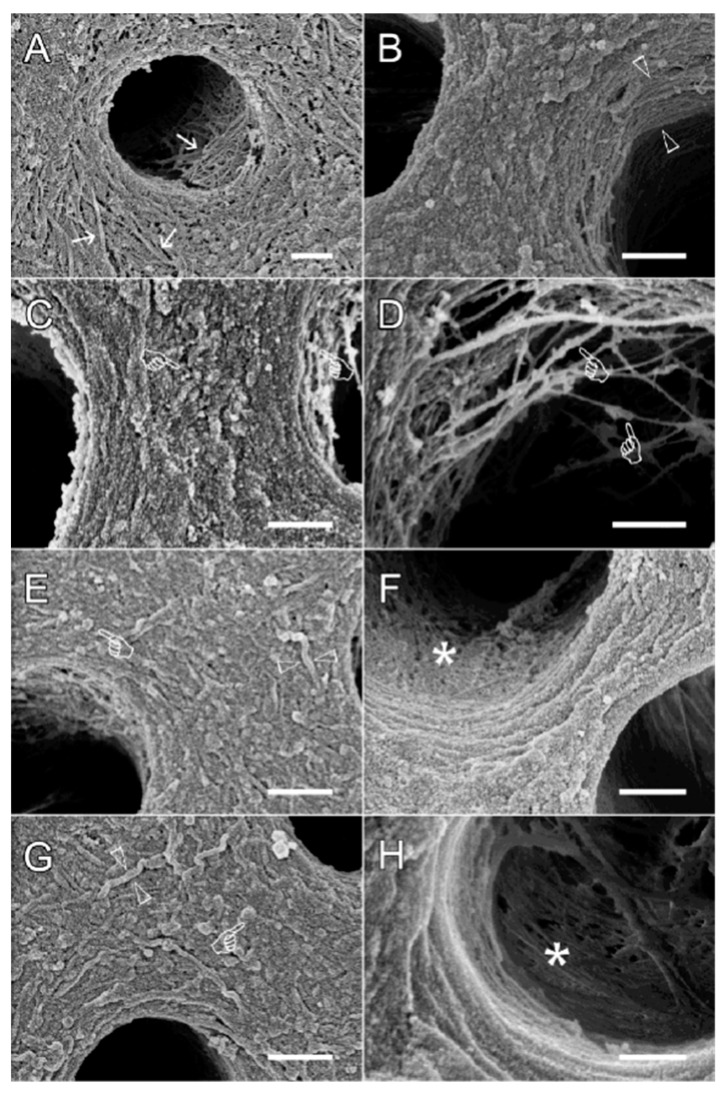
Representative SEM micrographs of the etched dentin resulting from different biomodification procedures. Images showing an illustrative area of the dentin surface of (**A**): DW group, intertubular and peritubular dentin collagen fibers (white arrows), different diameters of dentin collagen fibers (triangular arrows); (**B**): EDC group; (**C**,**D**): 1 mg/mL TMC + EDC group, TMC particles (finger); (**E**,**F**): 5 mg/mL TMC + EDC group, TMC particles (finger), collagen fibers (triangular arrow); (**G**,**H**): 10 mg/mL TMC + EDC group, membrane-like structure (asterisk). Scale bar = 500 nm.

Figure 2 shows the results of the scanning electron microscopy of the dentin collagen fibrillar network of the modified TMC and control specimens. The resulting structure of the collagen network showed that the TMC-treated specimen had fewer collagen gaps and a tighter collagen arrangement, while the untreated group had a looser collagen arrangement. In the DW group and EDC group (Figure 2A,B), the dentin collagen fibers exhibited a loose arrangement. After the addition of TMC and EDC, TMC particles could be observed on the collagen surface. Due to dehydration, we could see the collagen fibers separated from the tube wall (Figure 2D). As the concentration of TMC and EDC increased, we could observe clearer TMC particles, and the diameters of the TMC particles were 30–50 nm when the concentration of TMC and EDC was 1 mg/mL. The diameter of TMC particles increased to 120–150 nm when the concentration was increased to 5 mg/mL, and the diameter of collagen fibers increased to 150 nm. Meanwhile, with the increase in TMC content, TMC-collagen tended to form a membrane-like structure (Figure 2H, asterisk). The structure of collagen fibrils modified with TMC and EDC makes the possibility of the formation of micromechanical interlocking junctions increase, which can be effective in increasing the bond strength.

### 3.2. Assessment of TMC Adsorption to Collagen Surfaces

**Figure 3:**
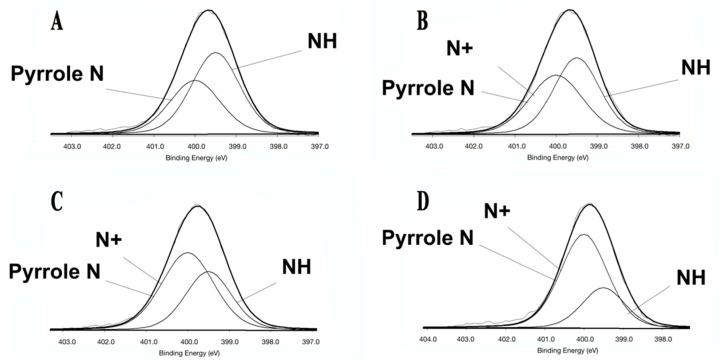
Adsorbed content on the collagen surface after TMC treatment detected by XPS; (**A**): DW Group; (**B**): 1 mg/mL TMC + EDC Group; (**C**): 5 mg/mL TMC + EDC Group; (**D**): 10 mg/mL TMC + EDC Group; The presence of peaks at binding energies of 400 and 399.5, where the peaks at 399.5 are -NH and -NH_2_ in collagen, replaced by NH in Figure 3, and N^+^ in quaternary ammonium salts and N in the pyrrole ring of collagen at 400 binding energy. The proportion of peaks at 400 increases with an increasing concentration of TMC.

The adsorbed content on the collagen surface after TMC treatment was determined by X-ray photoelectron spectrometry, and the results are shown in Figure 3. Since collagen contains a pyrrole ring and its binding energy is the same as that of the quaternary ammonium salt RNH^4+^, N^+^ has the same peak as pyrrole N (binding energy: quaternary ammonium salt RNH^4+^ = 400, pyrrole N = 400, amidogen N = 399). A linear relationship between the ratios of N^+^ in TMC and the various N in collagen can be observed with increasing concentration (R^2^ = 0.97759), demonstrating that the adsorption of TMC increases with increasing concentration. (Quaternary ammonium salt N^+^/Total N^+^: DW = 0; 1 mg/mL TMC + EDC = 0.111277505; 5 mg/mL TMC + EDC = 0.324478386; 10 mg/mL TMC + EDC = 0.521068708).

### 3.3. Dentin Collagen Degradation

**Figure 4:**
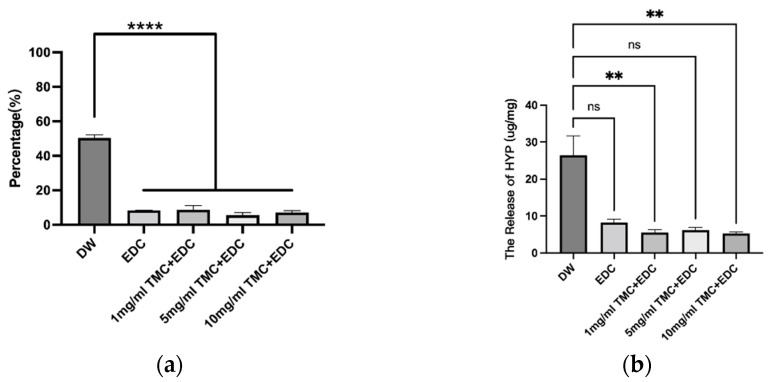
(**a**) The percentage of dry weight loss in each group; (**b**) The release of HYP in each group. (ns, no statistical significance; **, *p* < 0.01; ****, *p* < 0.0001).

By monitoring the amount of free hydroxyproline (HYP) in each group when the cross-linked collagen film was exposed to collagenase, it was possible to determine how resistant collagen was to enzymatic degradation after cross-linking with various agents in each group. The percentages of dry weight loss before and after degradation by collagenase for samples treated with different reagents are presented in Figure 4a, and the release rate of hydroxyproline after degradation is presented in Figure 4b. The samples treated with different concentrations of TMC and EDC together reduced the dry weight loss (*p* < 0.05), but there was no difference between the TMC groups and their application with EDC alone (*p* > 0.05); the same was true for the hydroxyproline release rate.

As shown in Figure 4a, in the DW group, which was not treated with any cross-linking agent, the dry weight loss of dentin collagen after enzymatic hydrolysis by collagenase reached 50.42%, and the release rate of HYP reached 26.45%. In the other four groups treated with the cross-linking agent, the values were 8.36% and 8.21% (EDC), 8.75% and 5.52% (1 mg/mL TMC + EDC), 5.54% and 6.13% (5 mg/mL TMC + EDC), and 7.13% and 5.28% (10 mg/mL TMC + EDC), respectively. The results showed that there were significant differences between the treatment group and the untreated group (*p* < 0.05).

### 3.4. Modulus of Elasticity

**Figure 5:**
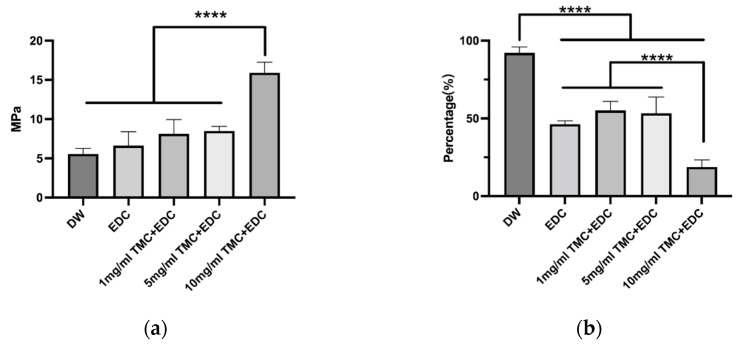
(**a**) Elastic modulus of each group after different treatment methods; (**b**) Degradation percentage of elastic modulus after collagenase degradation. (****, *p* < 0.0001.)

The results of the elastic modulus size of dentin collagen treated with different treatments are shown in Figure 5a. The difference in the elastic modulus group size between the groups was not statistically significant (*p* > 0.05), except for the enhancement in the 10 mg/mL TMC + EDC group, which was statistically significant (*p* < 0.05), and only the high concentration of TMC treatment was able to increase its elasticity. The loss of modulus of elasticity after degradation is shown in Figure 5b. Only the high concentration group of TMC can significantly reduce the loss of modulus of elasticity (*p* < 0.05).

### 3.5. Antibacterial Properties

**Figure 6:**
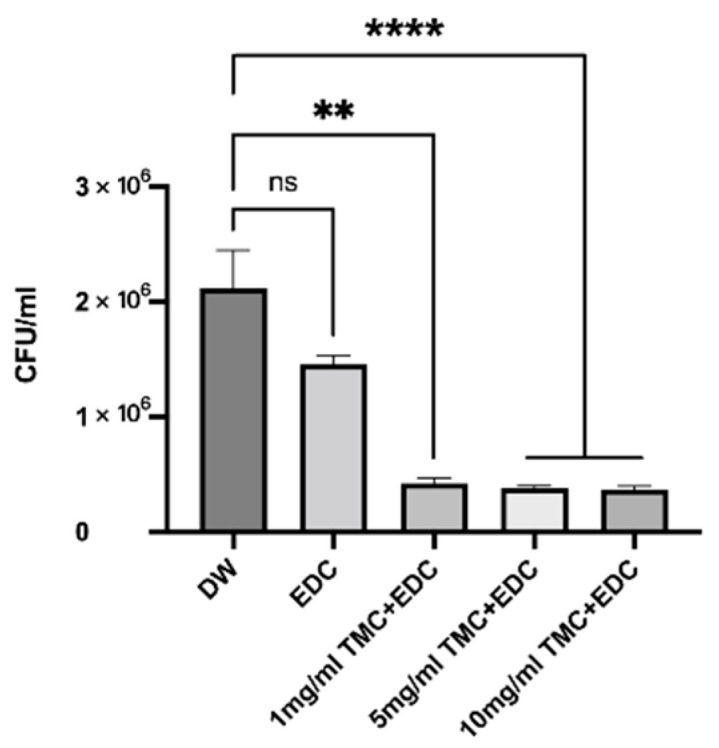
Colony-forming units of bacteria derived from biofilms on the dentin collagen matrix. (ns, no statistical significance; **, *p* < 0.01; ****, *p* < 0.0001.).

**Figure 7:**
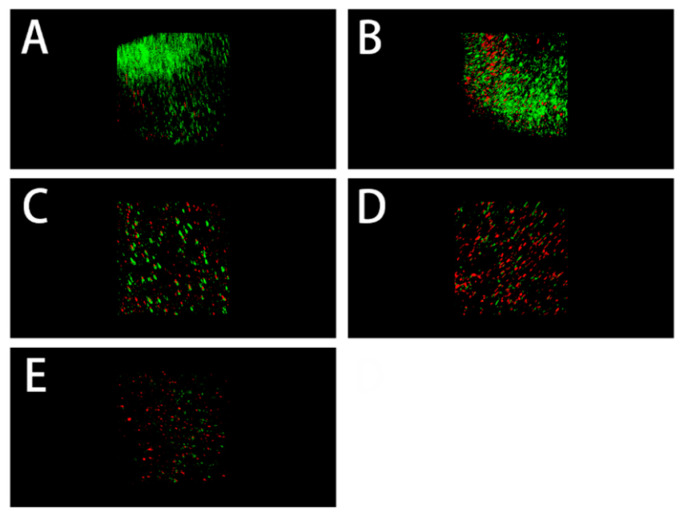
Representative CLSM images of *E. faecalis* biofilms in dentin tubules of different treatments, (**A**): DW; (**B**): EDC; (**C**): 1 mg/mL TMC + EDC; (**D**): 5 mg/mL TMC + EDC; (**E**): 10 mg/mL TMC + EDC.

The antimicrobial effect of collagen after cross-linking is shown in Figure 6 and Figure 7, where different concentrations of TMC were able to kill bacteria and inhibit bacterial growth (*p* < 0.05) while reducing the total biomass, but there was no difference between the TMC groups (*p* > 0.05). EDC alone was not able to kill bacteria (*p* > 0.05) and form biofilm structures, similar to the DW group.

## 4. Discussion

Fibrillar collagen is a strong and elastic biomaterial arranged in a highly organized hierarchy [35,36]. Type I collagen, the most prevalent of all collagen types, is a coiled helical trimeric molecule made up of repeating sequences of the amino acids Gly-XY, where X and Y are typically found as proline and hydroxyproline [16]. The final posttranslational modification of collagen is intermolecular cross-linking, which is the basis for the stability, tensile strength, and viscoelasticity of collagenous protofibrils [16]. Matrix metalloproteinases and cysteine proteases are the main causes of collagen degradation, and the binding sites for matrix metalloproteinases (MMPs) are located in the narrow 0.5-nm-wide fissures of collagen [37]. Thus, to degrade collagen, MMPs must bind to the collagen and unravel the structure of the collagen molecule, allowing the active site of the enzyme to react and attack specific glycine isoleucine peptide bonds in the peptide chain, ultimately leading to the cleavage break of the collagen peptide [38,39,40,41].

The use of EDC as a dentin collagen cross-linking agent has been extensively studied, and a study by Bedran-Russo et al. found that the application of EDC enhanced the mechanical properties of dentin collagen and the resin-dentin bond strength and stability of the adhesive interface over time [25,42]. However, a study by Mazzoni et al. found that EDC did not improve the resin-dentin bond strength when it was treated for only 1 min [43]. EDC, in its role in preventing collagen degradation, not only enhances collagen properties but also, more importantly, inhibits the activity of endogenous proteases. Mazzoni et al. found by enzyme profiling that the pretreatment of dentin collagen with only 0.3 M EDC completely inhibited protease activity [43], and A. Tezvergil-Mutluay found that even 0.01 M EDC inhibited rhMMP-9 activity by more than 99% [44]; these findings were also verified by Bedran-Russo et al. [45]. Moreover, EDC binds more rapidly with enzymes [44], thereby altering the conformation of the enzyme and inactivating it [34].

Chitosan is a (1–4) 2-amino-2-deoxy-β-glucan, chitin-derived polysaccharide with characteristics similar to those of glycosaminoglycans [46]. In the natural extracellular matrix, as an extracellular matrix-like substance, it increases the amine reaction sites, providing more cross-linking points for proteoglycans and glycosaminoglycans intertwining with fibrils of collagen to form ionic complexes, resulting in substantial stability of the fibrillar collagen network and giving it mechanical stability and considerable compressive strength [29,47]. Moreover, chitosan is insoluble in water in neutral or alkaline environments and only soluble in water under acidic conditions. Acid-etching of dentin in just the right acidic environment can make chitosan more soluble, thus increasing the number of free amine groups and making it easier to participate in covalent cross-linking [48]. A study by Tan et al. found that ultrastructural examination revealed small chitosan beads tightly bound to the collagen fiber backbone, and several collagen fibers or different parts of one fiber appeared to be woven and linked together by chitosan. The microstructure produced by the addition of chitosan prevents the collagen fibers from moving, induces the formation of interchain cross-links, and strengthens the fibers by anchoring them in their proper position. Collagen-chitosan interactions also affect the pore density and pore size of the matrix. Higher concentrations of chitosan in the matrix result in lower pore densities and smaller matrix pore sizes, and larger amounts of chitosan beads result in collagen fibers clustering together and filling the intersection within the fiber structure. This high-density microstructure stabilizes the porous collagen structure and helps maintain the stability of the matrix microstructure [49]. It is also more likely that the extended chitosan chains have the ability to wrap around the collagen helix structure, further enhancing the degradation resistance of collagen. Although chitosan has a good role in enhancing collagen properties and has been incorporated into collagen to improve its biological and mechanical properties [29], it has been less studied in oral materials, and Daood et al. found that excessive chitosan aggregation and formation within the collagen fiber structure exhibited detrimental effects on resin infiltration and mixed layers [50]. A. Shrestha et al. found that the application of carboxymethyl chitosan reduced the degradation of collagen and increased its ultimate tensile strength [51].

The EDC group was not examined by X-ray photoelectron spectroscopy because EDC is a zero-length cross-linker and would be washed off by deionized water and would not adsorb to the collagen surface. The results showed a linear correlation between the amount of adsorption on the collagen surface and the concentration of TMC used, with a peak at 400 binding energy in the blank group due to the presence of pyrrole structures in the tryptophan and proline and hydroxyproline in the collagen [52], which also has a binding energy of 400 for N, which suggests that TMC has successfully connected to the collagen of dentin. The elastic modulus of dentin collagen increased significantly only at the highest concentration of TMC, probably because the N binding energy in the biosynthesis of dentin collagen was not significant in the blank group. Extensive lysyloxidase-mediated covalent cross-linking already exists during the biosynthesis of dentin collagen [53], making collagen have good mechanical properties even without cross-linker treatment. Therefore, the addition of less TMC and EDC cross-linking to enhance the mechanical properties of dentin collagen is inherently difficult. Although the application of less TMC and EDC could not enhance the mechanical properties of dentin collagen, it could reduce the loss of elastic modulus, and the high concentration of the TMC treatment group was more effective than the other cross-linked groups, which may be attributed to the capacity of quaternary ammonium, inhibiting the matrix (MMPs) and enhancing the bonding durability [54]. Meanwhile, the application of TMC and EDC could reduce the degradation of dentin collagen [55,56], and the dry weight loss and hydroxyproline release rate of dentin collagen degraded by collagenase in this experiment were significantly lower than those of the uncross-linked group. The dry weight loss and hydroxyproline release rate of dentin collagen degraded by collagenase in this experiment were significantly lower than those of the uncross-linked blank group. However, the combined application of TMC and EDC was not significantly higher than that of EDC alone, and the different concentrations of TMC did not show differences.

Meanwhile, EDC, as a zero-length cross-linking agent, does not itself participate in cross-linking to dentin collagen, but it will be completely washed out during the rinsing process, so the use of EDC does not affect the antibacterial performance of chitosan and quaternary ammonium salts. The results of this experiment showed that TMC showed significant differences in antimicrobial performance with both the blank and EDC groups, which, because of the quaternary ammonium salts’ excellent antimicrobial properties, have long been used in restorative dentistry, and low concentrations of TMC (1 mg/mL) had already showed good antimicrobial performance, but the antimicrobial effect did not present a significant difference within all three TMC groups. At the same time, 12-Methacryloyloxy dodecylpyridinium bromide (MDPB) and benzalkonium chloride (BAC) are the two main types used in restorative materials [57]. Both have been shown to inhibit the activity of the endogenous proteases MMP-2, 8, and 9 and to significantly reduce the degradation of demineralized dentin collagen [57,58].

The limitation of the current study is that conclusions about the long-term antibacterial activity and collagenase degradation resistance cannot be made based on the findings of the 48-h research or the results of our experiment. Therefore, additional research is required to evaluate the antibacterial activity of biomodified dentin matrix, as well as the biostability.

## 5. Conclusions

Combined treatment of demineralized dentin with TMC and EDC significantly changed its morphological structure and reduced the dry weight loss and hydroxyproline release after degradation by collagenase. It was difficult to further enhance its elastic modulus but could reduce its elastic modulus loss; after the introduction of a quaternary ammonium functional group, dentin collagen showed significant antibacterial properties. However, there is no report on the long-term cross-linking performance and antimicrobial effect. Further studies should be performed to clarify the long-term effect of TMC in combination with EDC and to try to mix EDC and TMC together to reduce the clinical operation steps and the cross-linking processing time to adapt to the clinical treatment requirements and observe whether it is still effective.

## Data Availability

Not applicable.
